# Insights on Substitution Preference of Pb Ions in Sulfoaluminate Cement Clinker Phases

**DOI:** 10.3390/ma14010044

**Published:** 2020-12-24

**Authors:** Jianping Zhu, Yang Chen, Li Zhang, Kuo Yang, Xuemao Guan, Ruiqi Zhao

**Affiliations:** Henan Key Laboratory of Materials on Deep-Earth Engineering, School of Materials Science and Engineering, Henan Polytechnic University, Jiaozuo 454003, China; jianpingzhu@hpu.edu.cn (J.Z.); cy1102@126.com (Y.C.); zhangli_hpu@126.com (L.Z.); yangkuo04@163.com (K.Y.); guanxuemao@hpu.edu.cn (X.G.)

**Keywords:** sulfoaluminate cement clinker, solid waste, first-principles, Pb, electronic structure

## Abstract

The doping behaviors of Pb in sulfoaluminate cement (SAC) clinker phases were systematically studied combined with density functional theoretical simulations and experiments. The results present that, in the three composed minerals of C_4_A_3_S, C_2_S, and C_4_AF, Pb ions prefer to incorporate into C_4_A_3_S by substituting Ca ions. Further analyses from partial density of states, electron density difference, and local distortions show that such doping preference can be attributed to the small distortions as Pb introduced at Ca sites of C_4_A_3_S. The results and clear understandings on the doping behaviors of Pb ions may provide valuable information in guiding the synthesis of Pb-bearing SAC clinker, thus should draw broad interests in fields from sustainable production of cement and environmental protection.

## 1. Introduction

In recent years, with the urbanization, population growth, and industrialization, the amounts of solid waste are increasing sharply. It is estimated that 1.3 billion tons of waste is produced worldwide every year, and will increase to 3.4 billion tons by 2050 [1]. Solid waste, especially industrial by-product, contains many heavy metal elements, halogens, and other substances that are harmful to human beings. As a toxic heavy metal, plumbum (Pb), which exists broadly in fly ash (416.6 mg/g), sludge (92.5 mg/g), and coal dust (20.41 mg/g) [2], is extremely harmful to human health. How to effectively treat such industrial solid waste has become an essential issue of environmental protection.

Cement is the most widely used artificial product, which can be classified into various types according to the main composed phases, such as Portland cement (PC), sulfoaluminate cement (SAC), and magnesium phosphate cement (MPC) [3,4]. PC clinker mainly consists of C_3_S (3CaO·SiO_2_), C_2_S (2CaO·SiO_2_), C_3_A (3CaO·Al_2_O_3_), and C_4_AF (4CaO·Al_2_O_3_·Fe_2_O_3_) [5]. SAC clinker is composed of C_4_A_3_S (4CaO·3Al_2_O_3_·SO_3_), C_2_S, and C_4_AF [6]. The latter has high early strength, frost resistance, alkali resistance, and other excellent properties [7]. Therefore, SAC clinker may play key role in cement-related materials in future. It is reported that SAC clinker can be produced by utilizing solid waste as raw materials [8,9]. The co-processing of solid waste by cement kilns is an efficient way for safe disposal of heavy metals [10], and it is also the direction of sustainable development of the cement industry in the future. In the process of clinker formation, heavy metal ions can be solidified in clinker minerals by forming solid solutions, thus effectively avoid the formation of secondary pollution. Compared with Portland cement, heavy metal ions are more likely to dissolve into SAC clinker phases [11]. Moreover, the content of Pb in SAC clinkers produced by this technology is below the levels limited by the international standards [12]. Moreover, the firing temperature of SAC clinker could be reduced by 150 °C to 200 °C [13,14], which can reduce carbon emissions [15,16].

Several researchers studied the effects of Pb on the properties of SAC clinker as well as its hydration products. For example, Mao, Y. et al. used solid waste as raw materials and found that Pb can be dissolved in SAC clinker phases [17]. Ma, B. et al. reported that the solidification of Pb ions can increase the hydration rate of SAC clinker and the jelling time can be reduced 72% by introducing 2 wt% Pb(NO_3_)_2_ [18]. Wang. L. et al. reported the carbonation and water washing can effectively reduce the volatilization of Pb in the calcination process [19]. To better utilize Pb-bearing raw materials as well as SAC-based materials, it is essential to learn the existing state, the possible substitution preference, and the factors influencing doping behaviors of Pb ions in SAC clinker phases.

Besides the progress from experimental researchers, it also intrigued great interests of theorists. Especially in recent years, with the rapid development of parallel computing and simulation methods, it has become possible to simulate the complicated system of various cements [20,21,22,23,24]. Zhu, J. et al. studied the doping behaviors of Zn ions in PC clinker with density functional theoretical (DFT) simulations and experiments [20]. They found that Zn ions prefer to substitute Fe in C_4_AF and the extracted Fe will react with another phase, C_3_A. Zhao, R. et al. reported the doping preference of Mg ions in C_4_AF by substituting Fe, while in the other three minerals of PC clinker, they prefer to replace Ca ions and the solubility follows the sequence of C_3_S ≈ C_3_A > C_2_S [21]. Zhu, J. et al. studied the substituting behaviors of Ba in SAC clinker, the extinct Ba atoms prefer to incorporate into C_4_A_3_S by substituting Ca atoms while little enters the other two minerals, C_2_S and C_4_AF [22]. Li, N. et al. revealed the doping preference of Mn and Cu in PC clinker combined with DFT simulations and experiments [23,24]. In this work, we attempt to unveil the doping behaviors and existing form of Pb in SAC clinker phases, and based on these understandings to further evaluate the possibility of producing SAC clinker by utilizing Pb-bearing industrial solid waste.

## 2. Simulations and Experiments

### 2.1. Simulations

**Figure 1:**
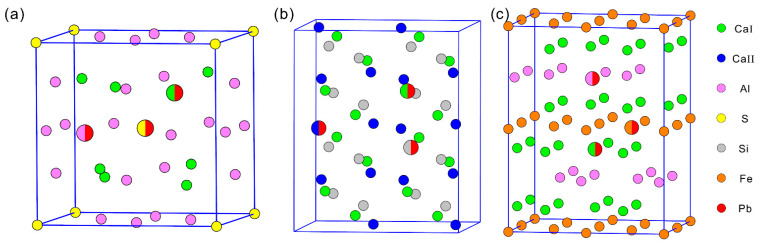
Schematic structures of SAC clinker phases: (**a**) C_4_A_3_S, (**b**) C_2_S, and (**c**) C_4_AF. The doping sites are highlighted with bigger bicolored balls in each panel. One color (the red one) represents Pb and the other one stands for the atoms been substituted. The green, blue, purple, yellow, grey, orange, and red balls represent six-fold Ca, eight-fold Ca, Al, S, Si, Fe, and Pb, respectively.

The mineral C_4_A_3_S, which accounts for about 55–75 wt% of SAC clinker, has cubic, tetragonal, and orthorhombic forms. Here, the cubic form was used to model C_4_A_3_S [25]. C_2_S also possesses several crystal structures and the β-form was used as the matrix to model C_2_S as it can be retained at room temperature. [26,27]. Considering the complexity and poor symmetry of β-C_2_S, the Ca ions with 6- and 8- fold coordination numbers are denoted as CaI and CaII, respectively. The ferrite phase usually exists as continuous solid solutions, and the one with equal molar aluminum and iron, C_4_AF, is used to model tetracalcium aluminoferrite [28]. To reduce the influence between adjacent doping atoms and ensure the comparability of clinker with similar content, primitive cell (C_4_A_3_S) and the supercells of 2 × 2 × 1 (for β-C_2_S) and 2 × 1 × 2 (for C_4_AF) were used to model doping behaviors of Pb in SAC clinker phases. The detailed schematic structure models of three minerals are shown in Figure 1.

DFT simulations were performed with the Vienna Ab-initio Simulation Package (VASP) in this work [29,30], and the generalized gradient approximation (GGA) within Perdew–Burke–Ernzerhof (PBE) functionals were adopted as the exchange-correlation potential [31]. The energy cutoff was 500 eV. The K-points were sampled with Gamma-centered scheme in the first Brillouin zone [32]. The K-point meshes with density of 0.02 and 0.01 were used for configuration relax and calculations of partial density of states (PDOS), respectively. The detailed K-points are summarized in Appendix A Table A1. The convergence criteria of 10^−5^ eV/atom and 0.01 eV/Å were used for the energy and force, respectively. The electron density difference (EDD) was calculated by CASTEP [33]. The models and results were visualized by VESTA.

The normalized defect formation energy (${\bar{E}}_{f}$) was used to characterize the possibility of substitution, which can be defined as [34,35]:

(1) $${\bar{E}}_{f}=\frac{E-E_{0}+\mu_{\mathrm{Pb}}-\mu_{X}}{\omega }$$

where *E* and *E*_0_ are the energies of the doped and original clinker phases, respectively; *μ*_Pb_ and *μ*_X_ are the chemical potentials of Pb and the atoms, X (X = Ca, Al, S, Si and Fe), respectively. The values of *μ*_Pb_ and *μ*_X_ can be calculated from corresponding bulk materials [36]. More details about the bulk materials and their chemical potentials are summarized in Table A2. ω is the mass concentration of Pb in each phase.

### 2.2. Experiments

Both pristine and Pb-doped SAC clinker samples were prepared to learn the doping behaviors of Pb ions. The analytical grade materials CaCO_3_, CaSO_4_, SiO_2_, Al_2_O_3_ and Fe_2_O_3_ were used as raw materials. The mass of these materials was set as constants to ensure the compositions of each mineral of SAC clinker. The potential mineral compositions calculated from Bogue equations [37] are C_4_A_3_S (56 wt%), C_2_S (29 wt%) and C_4_AF (15 wt%), respectively. Different amounts of PbO_2_ were added as Pb source. For comparison, the samples of pristine and Pb-doped C_4_A_3_S were also prepared. The detailed ratios of raw materials are summarized in Table 1.

The mixture of all materials used to prepare each sample was grinded for 1 h in agate mortar and pressed into pellets (height, ~10 mm; diameter, ~30 mm) at a pressure of 30 MPa. The obtained pellets were put in alumina crucibles and sintered for 30 min at 900 °C and 3 h at 1350 °C with a heating rate of 10 °C/min. All samples were quenched to room temperature in air. The obtained samples were crushed into small pieces. Some were polished and sputter-coated with gold for backscattered electron (BSE) measurement. The other parts and the C_4_A_3_S pellets were ground until passing 200 mesh sieves for X-ray diffraction (XRD) measurements.

BSE measurements were performed with the scanning electron microscopy (SEM, Merlin Compact, Carl Zeiss NTS GmbH, Oberkochen, Germany) coupled with the energy-dispersive spectroscopy (EDS, Oxford, UK) at 15 kV. XRD measurements were carried out using Rigaku diffractometer (XRD, Smart lab, Rigaku, Tokyo, Japan). The data were collected from 5° to 80° with a step size of 5°/min and the counting time of 0.02° per step under 40 kV and 150 mA.

## 3. Results and Discussion

### 3.1. Defect Formation Energies

**Figure 2:**
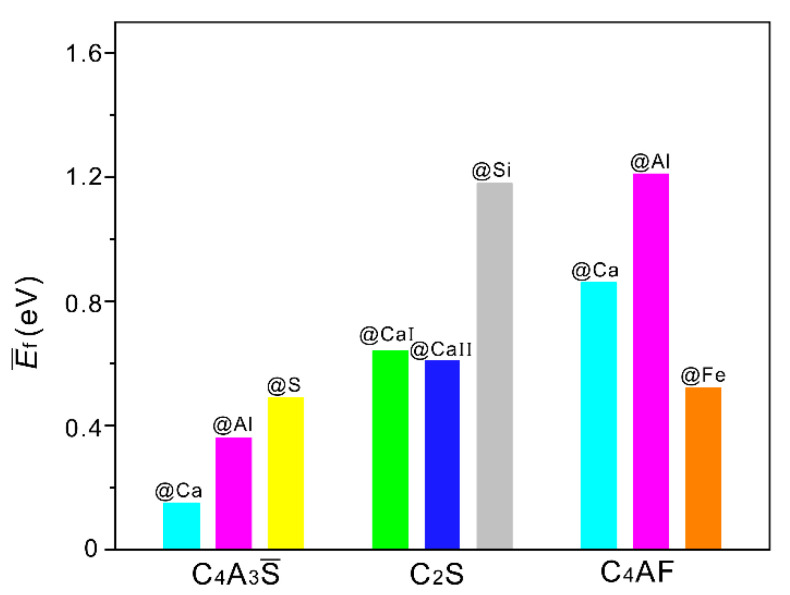
The normalized defect formation energies (${\bar{E}}_{f}$) of configurations with Pb introduced at different sites.

The configurations of three minerals with Pb ions introduced at different sites are shown in Figure 1. The doping sites were highlighted by bigger bicolored balls. The values of ${\bar{E}}_{f}$ can be used to characterize the possibility of forming solid solutions. Generally, the smaller values mean the higher possibilities of reaction. Figure 2 shows ${\bar{E}}_{f}$ of all configurations of SAC clinker phases (see Figure 1 for detailed geometries). It can be seen that the values of all ${\bar{E}}_{f}$ are positive. Additionally, the configuration with Pb introduced at Ca site of C_4_A_3_S has the lowest ${\bar{E}}_{f}$, indicating that Pb tends to incorporate into C_4_A_3_S by substituting Ca. The positive ${\bar{E}}_{f}$ implies that extra energy is required to overcome the reaction barrier. As SAC clinker was prepared at 1350 °C, such barrier may be easily overcome under the above synthetic conditions.

### 3.2. Experimental Analyses

**Figure 3:**
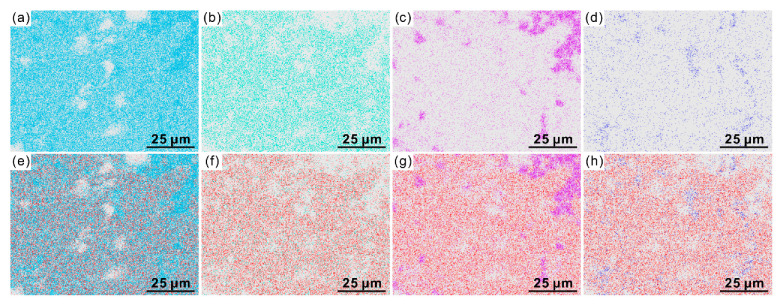
(**a**–**d**) The element distributions of Ca (**a**), S (**b**), Si (**c**), Fe (**d**), and (**e**–**h**) overlaps of Pb with the above four elements in SAC clinker samples.

In order to confirm the doping preference of Pb in the SAC clinker, several samples with Pb content of 0 wt%, 11.82 wt%, 17.74 wt%, and 23.65 wt% were prepared, respectively. The SEM-EDS of elements including Ca, S, Si, and Fe of the sample A4 are shown in Figure 3a–d, respectively. The brighter regions represent those with high content of corresponding elements. The distribution regions of Ca are contributed by all composed minerals of SAC clinker while those of S, Si, and Fe are solely originated from the mineral C_4_A_3_S, C_2_S and C_4_AF, respectively. Therefore, the regions of S, Si, and Fe represent the zones of corresponding minerals. To clearly show the possible doping preference of Pb in three minerals, the distributions of Pb were overlapped with those of Ca, S, Si, and Fe, respectively. The obtained figures are shown in Figure 3e–h, respectively. It can be seen from Figure 3e that the regions of Ca are larger than that of Pb, indicating that Pb ions prefer to locate in certain phases. Careful examinations of panels Figure 3f–h present that the distributions of Pb consist almost exactly with that of S (see panel b and f) while present poor overlap with those bright regions of Si (panel c and g) and Fe (panel d and h). Therefore, Pb ions should mainly incorporate into the mineral C_4_A_3_S. Due to the limitations of EDS, it is hard to judge which element is the one that Pb ions tend to substitute. Thus, we analyzed the XRD results in the following part.

**Figure 4:**
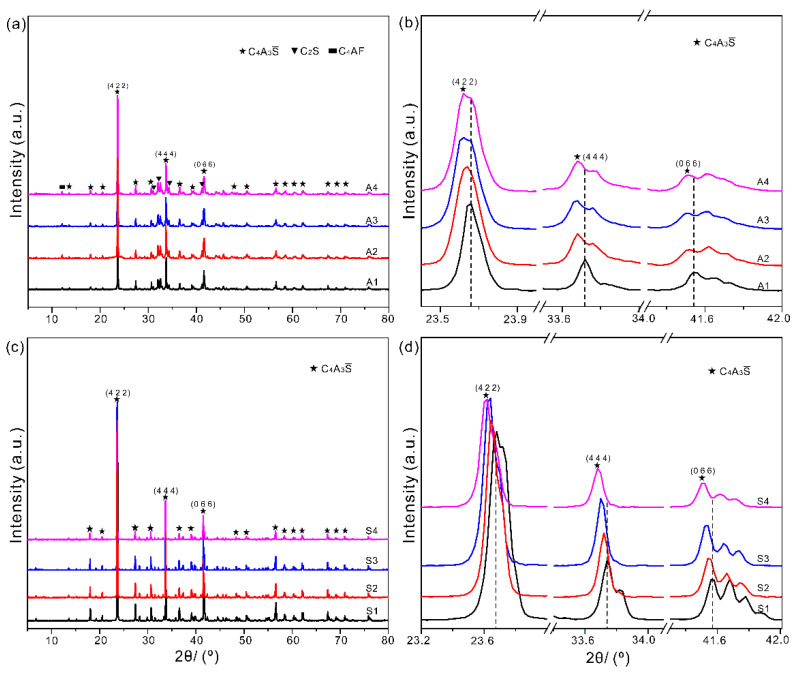
The full ranged and enlarged XRD patterns of SAC clinker (**a**,**b**) and the single mineral C_4_A_3_S (**c**,**d**). The Miller indices of three main peaks of C_4_A_3_S are also presented in each panel.

The full ranged XRD patterns of SAC clinker with different dosages of PbO_2_ are shown in Figure 4a. All diffractions were assigned according to the standard diffraction patterns of C_4_A_3_S (PDF#-71-0969), C_2_S (PDF#-33-0302), and C_4_AF (PDF#-71-0607). The peaks belonging to the same phase are identified with identical symbols. From Figure 4a, we can see that the peaks belonging to C_4_A_3_S are much higher than those of C_2_S and C_4_AF, which can be attributed to the predominant content of C_4_A_3_S in the prepared SAC clinker. The Miller indices of three main peaks of C_4_A_3_S are also labelled in Figure 4a. To present the variations as Pb introduced, the enlarged parts of these three main peaks are shown in Figure 4b. It clearly presents that these three peaks continuously shift towards left as the dosage of Pb increases, indicating that Pb ions successfully incorporate into C_4_A_3_S. According to Bragg equation, the decrease in Bragg angles means the increase in lattice constant as Pb is introduced in SAC clinker.

To further verify the incorporation possibility of Pb ions in the mineral C_4_A_3_S, both pristine and Pb-doped C_4_A_3_S were also prepared. The full ranged and enlarged XRD patterns of C_4_A_3_S are shown in Figure 4c,d, respectively. The peaks as well as the Miller indices of the three main peaks are also presented in Figure 4c,d. The left shifts observed in the single mineral C_4_A_3_S is consistent with those from XRD patterns of SAC clinker.

Both the above element distributions and XRD patterns show that Pb ions prefer to incorporate into the mineral C_4_A_3_S. However, more quantitative experiments and further analyses are needed to determine the exact solubility of Pb ions in SAC clinker in the future. Besides that, there are several sites, such as those occupied by Ca, Al, and/or S, can host Pb ions. As shown in Table A3, the ionic radius of these ions is smaller than that of Pb ions. In other words, the incorporation of Pb in the above sites can result in increase in lattice parameters. The exact sites of Pb in C_4_A_3_S cannot be determined from experiments. To further figure out the exact doping sites, we analyzed the partial density of states and electron density difference of all minerals before/after Pb is introduced.

### 3.3. Partial Density of States and Electron Density Difference

**Figure 5:**
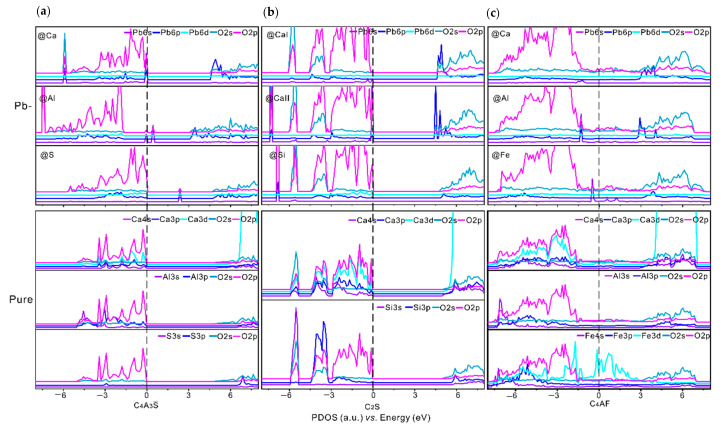
The partial density of states (PDOS) of pure and Pb-doped SAC clinker Phases: (**a**) C_4_A_3_S, (**b**) C_2_S, and (**c**) C_4_AF. The Fermi energy was set to zero eV.

The PDOS of pristine and Pb-doped C_4_A_3_S, C_2_S and C_4_AF are shown in Figure 5. Compared with pristine C_4_A_3_S, in configuration with Pb introduced at Ca, the newly formed Pb-O bonds are mainly contributed by the overlaps of electron density on Pb-6*s,* Pb-6*p* and O-2*p* orbitals. For configurations with Pb introduced at Al and S, the electron density of states of Pb-6*s* possess higher energies and present little overlaps with that of O-2*p* orbitals, indicating weaker Pb-O bonds in configurations with Pb introduced at Al and S. For the minerals C_2_S and C_4_AF, all the newly formed Pb-O bonds present much less overlaps between Pb-6*s*, Pb-6*p* and O-2*p* orbitals. Thus, Pb should prefer to substitute the Ca atom in C_4_A_3_S.

**Figure 6:**
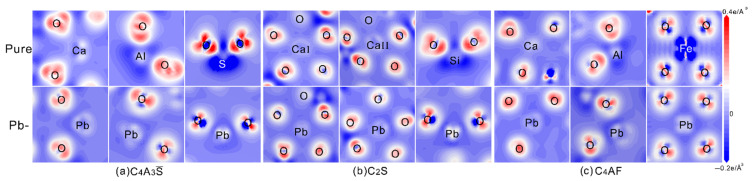
Electron density difference (EDD) of pristine and Pb-doped minerals of SAC clinker: (**a**) C_4_A_3_S, (**b**) C_2_S and (**c**) C_4_AF. The blue and red isosurfaces stand for electron accumulation and reduction, respectively.

EDD can visually show the electron redistributions between adjacent atoms, thus can be used to determine the strength of bonds. The EDD results of pristine and Pb-doped C_4_A_3_S, C_2_S, and C_4_AF are shown in Figure 6a–c, respectively. The central ions and partial oxygen atoms are also presented in each panel. The red and blue regions stand for accumulation and reduction of electrons, respectively. In all configurations, the regions around O present red due to its most strong electronegativity. The regions around Al and Si present similar electron distributions due to their similar electronegativity, while the regions with S and Fe as centered atoms present obviously blue, which can be attributed to their predominant numbers in valent electrons. For regions centered with Ca and Pb, they present light blue, indicating much less electron transfer occurs from Ca/Pb to O. In configurations with Pb introduced at Al, S, Si, and Fe, the Pb-centered regions present lighter blue than those in pristine minerals. For the configurations with Pb introduced at Ca, the one in C_4_A_3_S present a little deeper blue than that in C_2_S and C_4_AF. Thus, Pb ions should prefer to occupy the sites of Ca in C_4_A_3_S, well consistent with the results obtained from PDOS.

### 3.4. Local Structural Distortions

**Figure 7:**
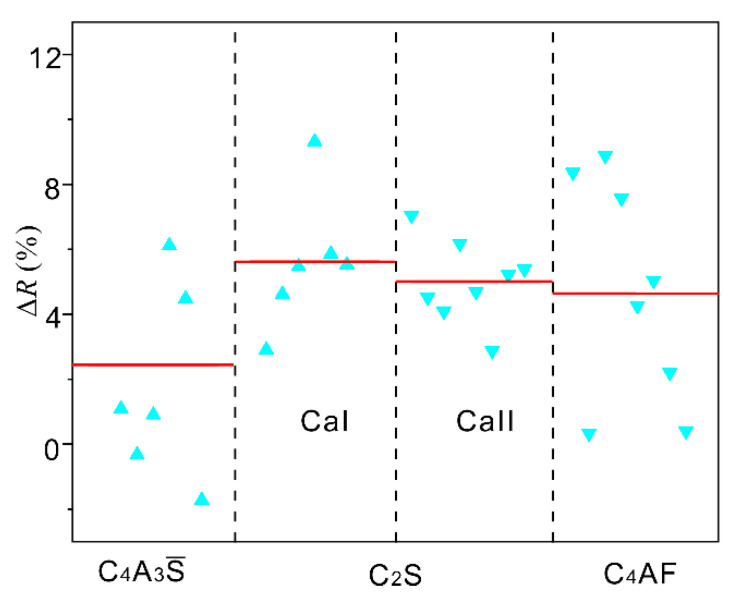
The relative bond length (∆*R*) of C_4_A_3_S, C_2_S, and C_4_AF with Pb ions introduced at Ca sites. The red lines represent the averaged values of ∆*R*. The regular and the inverted triangles represent the 6- and 8-fold Ca, respectively.

The above results present that, although all three minerals contain Ca atoms, Pb ions prefer to substitute the Ca atoms in C_4_A_3_S To further determine the preferred incorporation, the local distortions of Pb-centered polyhedrons were carefully analyzed. Here, we use the relative bond length ∆*R*, (∆*R* = (*R* − *R*_0_)/*R*_0_ × 100%; *R* and *R*_0_ represent the bond length of Pb-O and Ca-O, respectively) to characterize the local distortions. The results are shown in Figure 7. The values of ∆*R* fall in a large range of ~−2–10% in three minerals. For a reference, the averaged ∆*R* is also presented as red lines in Figure 7. Among these configurations, the mineral C_4_A_3_S presents the smallest distortions as Pb is introduced. The small distortions in C_4_A_3_S may also play positive roles in hosting Pb ions.

## 4. Conclusions

In this work, we systematically studied the possibility and doping preference of Pb in SAC clinker phases by DFT simulations and experiments. Our results show that Pb ions can incorporate into SAC clinker by forming solid solutions. Such incorporation probably can be attributed to the preferred substitution of Pb at Ca sites of C_4_A_3_S. The preferred substitution can be attributed to the small structure distortions between Pb-O and Ca-O as Pb ions are introduced. The understandings on the existing state and the doping preference of Pb could provide valuable information in the synthesis of SAC clinker by utilizing Pb-bearing solid waste, thus is very meaningful both in sustainable development of SAC industry and environment protections.

## Figures and Tables

**Table 1:** The ratios of materials used to prepare pristine and Pb-doped SAC clinker and the mineral C_4_A_3_S.

Samples		Ratio of Materials (wt%)					
		CaCO_3_	SiO_2_	Al_2_O_3_	CaSO_4_	Fe_2_O_3_	PbO_2_ *
SAC	A1	54.56	7.66	23.58	10.46	3.74	0
	A2	54.56	7.66	23.58	10.46	3.74	11.82
	A3	54.56	7.66	23.58	10.46	3.74	17.74
	A4	54.56	7.66	23.58	10.46	3.74	23.65
C_4_A_3_S	S1	40.47	-	40.80	18.73	-	0
	S2	40.47	-	40.80	18.73	-	16.86
	S3	40.47	-	40.80	18.73	-	25.29
	S4	40.47	-	40.80	18.73	-	33.71

* Notes: The dosage of PbO_2_ is calculated based on the masses of products after calcination.

## Data Availability

Data available in a publicly accessible repository.

## Appendix A

**Table A1:** Minerals, supercells, lattice parameters, and detailed k point meshes used for relax and partial density of states (PDOS).

Minerals	Supercells	Lattice Parameters/Å	Relax	PDOS
C_4_A_3_S [25]	1 × 1 × 1	*a* = *b* = *c* = 9.19	3 × 3 × 3	5 × 5 × 5
*β*-C_2_S [26]	2 × 2 × 1	*a* = 11.01, *b* = 13.51, *c* = 9.31	2 × 2 × 3	5 × 4 × 5
C_4_AF [28]	2 × 1 × 2	*a* = 11.17, *b* = 14.60, *c* = 10.75	2 × 2 × 2	5 × 3 × 5

**Table A2:** Phases, space group, ICSD IDs, lattice parameters [36], and chemical potentials (μ) of Pb and the atoms that have been replaced by Pb.

Phase	Space Group	ICSD IDs	Lattice Parameters						*μ*/eV
			a/Å	b/Å	c/Å	α/°	β/°	γ/°	
αPb	Fm3m	648,343	0.50	0.50	0.50	90	90	90	−3.56
αCa	Fm3m	426,932	5.59	5.59	5.59	90	90	90	−1.92
αAl	Fm3m	53,774	4.05	4.05	4.05	90	90	90	−3.75
αS	P2/c	82,372	13.64	9.25	10.90	57	90	90	−4.12
αSi	Fd3m	181,356	5.43	5.43	5.43	90	90	90	−5.43
αFe	Im3m	631,729	2.87	2.87	2.87	90	90	90	−8.24

**Table A3:** Ions, coordination number, electronegativity, and ionic radius [38] of Pb and other ions that have been substituted.

Ions	Coordination	Electronegativity	Radius/Å
Pb^4+^	8	1.557	1.08
Ca^2+^	6	1.032	1.00
Al^3+^	4	1.499	0.39
S^6+^	4	2.479	0.12
Si^4+^	4	1.709	0.26
Fe^3+^	6	1.687	0.645
